# Effects of Climate and Water Limitation on Reproductive Traits and Trait Divergence Across Soil Boundaries in a Serpentine‐Tolerant Annual Herb

**DOI:** 10.1002/ece3.73785

**Published:** 2026-06-23

**Authors:** Nicole Ibañez, John Paul, Adelaide Ergastolo, Sevan Suni

**Affiliations:** ^1^ The University of San Francisco San Francisco California USA; ^2^ The Pollinator Partnership San Francisco California USA

**Keywords:** *Antirrhinum vexillocalyculatum*, drought, flowering time, functional traits, precipitation, reproductive traits, serpentine, trait divergence

## Abstract

Biodiversity hotspots are often found in areas with high precipitation or diverse microhabitats. For plants, geographic areas with high soil variability tend to have high species richness or functional trait diversity. Serpentine soil has driven the diversification of numerous lineages, and many taxa show phenotypic variability across soil types. Precipitation can increase effects of soil on divergence, but this has been examined only for growth and vegetative traits. Using a greenhouse common garden, we characterized reproductive phenology for the serpentine‐tolerant annual herb *Antirrhinum vexillocalyculatum* along a moisture gradient and evaluated whether trait divergence varies with precipitation. Furthermore, we conducted a water limitation experiment and examined whether water limitation changes trait expression or divergence in trait expression. Finally, through field observations we assessed whether reproductive phenology, divergence in reproductive phenology, or the potential for gene flow differs between wetter and drier areas. We found strong effects of precipitation regime on flowering phenology, and some support for the hypothesis that precipitation enhances divergence across soil types. Experimental water limitation reduced floral displays, caused phenological delays, and increased divergence across soil types for some traits. In the field, the proportion of plants flowering was consistently greater in the wetter area, and the potential for gene flow was higher from non‐serpentine to serpentine populations. Despite phenological differences among ecotypes, flowering distributions overlapped to a large extent along the precipitation cline and under water limitation. This suggests that gene flow across soil types is a dominant force in this system but may be lower in wetter areas and under future drought. Shortened flowering durations and floral displays under drought suggest that plant reproductive potential will decrease in the future, with the possibility of lower adaptive gene flow from serpentine to non‐serpentine populations should it occur.

## Graphical abstract

Soil and precipitation can drive divergence in plant populations, yet less is known about the potential for synergistic effects between soil and precipitation on reproductive traits. We characterized variation in flowering phenology across soil boundaries and along a precipitation gradient and experimentally evaluated how water limitation affects reproductive traits and trait divergence. We found strong effects of soil, precipitation, and water limitation on flowering phenology and divergence across soil types, suggesting that gene flow across soil types may be lower in wetter areas and drought may decrease gene flow across soil types in the future.

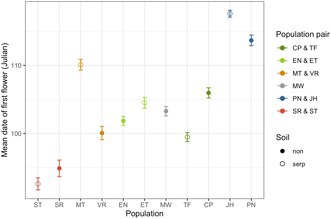

## 1 Introduction

Biodiversity is influenced by ecological factors that drive the evolutionary diversification, coexistence, or extinction of distinct lineages. Hotspots of diversity are often found in areas with high precipitation and a lack of temperature extremes (Hawkins et al. 2003; Field et al. 2009), or in areas with diverse microhabitats (Stein et al. 2014). For plants, edaphic variation is an important driver of diversification (Hulshof and Spasojevic 2020). This manifests as geographic areas with high soil variability tending to have high plant species richness (García et al. 2020; Hofmeister et al. 2024) or functional trait diversity (Hulshof and Spasojevic 2020).

Serpentine soils, in particular, have driven the diversification of many plant lineages globally (Anacker et al. 2011). Serpentine soils are ultramafic (high in Mg and Fe and low in silica), and they occur in patches on most continents where they have been uplifted by tectonic plate movement (Harrison 1997). They are important selective agents for plants due to their high amounts of heavy metals (Fe, Ni, Cr, and Co), high Mg:Ca ratios, and low levels of primary nutrients (P, K and N; Whittaker 1954). They are also often rockier than nearby soil types, which can result in lower water‐holding capacity relative to other soil types (Walker 1954; Kruckeberg 2002). Despite the harsh growing conditions that serpentine soils provide for plants, they often contain high species diversity relative to the geographic areas they cover. For example, in California, serpentine soils harbor as many as 10% of the endemic species found in the state, despite covering only 1% of the geographic area (Safford et al. 2005; Brady et al. 2005).

The evolution of serpentine endemism has been proposed to stem from a tradeoff between competitive ability and the expression of stress‐tolerance traits that enable life in serpentine soil (Kruckeberg 1951). Serpentine endemics tend to have traits that enable them to tolerate high amounts of heavy metals or habitat bareness, or to escape or avoid drought (Heschel et al. 2017; Rossington et al. 2018). Relative to sister taxa that grow on other soil types, serpentine endemics tend to have slower growth rates (Whittaker 1954; Brady et al. 2005), smaller specific leaf area (Samojedny Jr et al. 2022), and earlier flowering time (Brady et al. 2005; Dittmar and Schemske 2018; Lazzaro et al. 2021; but see Sianta and Kay 2021). These traits may make serpentine endemics poorer competitors in non‐serpentine soils than taxa that are not serpentine‐adapted (Jurjavcic et al. 2002; Fernandez‐Going and Harrison 2013; Cacho and Strauss 2014) and be responsible for the persistence of certain taxa only on serpentine soil.

In contrast to serpentine endemics, some plant species are able to live both on and off serpentine soil. These serpentine tolerators are edaphic generalists at the species level (Sianta and Kay 2019) and may maintain gene flow across soil boundaries (Arnold et al. 2016). However, they also often show evidence of genetically‐based trait differences across soil types (Arnold et al. 2016; Heschel et al. 2017; Sianta and Kay 2021). Similar to serpentine endemics, serpentine tolerator species growing on serpentine soil have been found to have more pronounced drought avoidance traits than those same species growing on non‐serpentine soil, such as low SLA (Fernandez‐Going et al. 2012; but see Adamidis et al. 2014), or leaf thickness (Adamidis et al. 2014). These trait differences may explain the finding that plant communities on serpentine soil are less sensitive to climate change than communities on non‐serpentine soil (Harrison et al. 2015).

Given that the intensity of drought is expected to increase in the coming decades (Soulé and Knapp 2024), traits that facilitate drought tolerance or avoidance may have fitness benefits for plants on non‐serpentine soil as well as on serpentine soil. Furthermore, it is possible that gene flow from serpentine to non‐serpentine populations could confer alleles that are adaptive under future climates. It is difficult to predict if adaptive gene flow across soil types may occur in the future because even if the underlying genetic architecture and allelic composition governing reproductive phenology are similar, traits can be expressed differently given edaphic conditions. Nevertheless, the majority of gene flow in plant populations occurs via the movement of pollen (Levin and Kerster 1974; Ennos 1994), so should it occur, gene flow between soil types would likely require phenotypic overlap in reproductive traits (such as flowering phenology) between serpentine and non‐serpentine populations.

Trait divergence between plants living on and off serpentine soil has been hypothesized to be greater in more productive climates due to the trade‐off between competitive ability and stress‐tolerance (Anacker and Harrison 2012). A field study that included both serpentine endemics and populations of tolerators on different soil types found greater divergence in growth‐related functional traits in field populations in more productive areas along a moisture cline (Fernandez‐Going et al. 2013). However, whether these patterns of divergence in trait expression were reflective of phenotypic plasticity or adaptive evolutionary processes was not evaluated. Evolutionary divergence is facilitated by the accumulation of pre‐zygotic reproductive isolating barriers such as flowering time differences (Lafon‐Placette et al. 2016). Differences in flowering time have been found between populations of tolerator species on and off serpentine, as well as between sister species pairs that included a serpentine endemic and the closest‐related non‐serpentine taxon (Sianta and Kay 2021). However, that study evaluated only single population pairs across species, so expanding the scope of these investigations to include many populations along a moisture cline may provide unique insight into the extent to which soil and climatic productivity mediate trait expression and reproductive divergence across soil types.

Here, we examined ecological drivers of reproductive traits, phenological divergence, and the potential for gene flow between serpentine and non‐serpentine populations of a serpentine tolerator along a productivity gradient. First, we asked if nearby populations on and off serpentine soil vary in flowering phenology, and whether the divergence in phenology is associated with moisture availability. We predicted that plants from serpentine sites and drier sites would flower earlier and that phenological divergence across soil types would be greater in areas with higher precipitation. Second, to gain insight into how these traits may change under future climates, we evaluated the extent to which flowering phenology is phenotypically plastic with regards to soil moisture and whether plasticity varies with soil type or precipitation regime. We predicted that plasticity would be higher for serpentine plants and those from drier areas, because higher plasticity has been shown to be associated with greater drought avoidance in other systems (Couso and Fernández 2012). Finally, we sought to gain insight into the potential for gene flow based on flowering phenology between field populations from different soil types, and understand if the potential for gene flow varies with precipitation regime. We predicted that the potential for gene flow would be higher in drier areas due to greater phenological overlap in those areas. To accomplish these objectives, we conducted a greenhouse common garden experiment in which we grew plants from seeds collected from paired serpentine and non‐serpentine populations along a moisture cline, subjected a subset of plants from each population to a limited‐moisture treatment, and characterized their flowering phenology. We also conducted field observations of flowering phenology for a subset of the populations from which seeds had been collected. This use of field observations coupled with an experimental greenhouse experiment allows us to evaluate the joint effects of soil and native moisture regime on flowering phenology while gaining insight into the extent to which phenotypes are driven by plasticity. In addition, our characterization of flowering phenology for nearby serpentine and non‐serpentine populations in the field allows us to calculate an idealized upper limit for the potential for gene flow and evaluate how this varies with native moisture regime.

## 2 Methods

### 2.1 Species and Sampling

We conducted our study in the California Floristic Province, which is a biodiversity hotspot that contains approximately 4500 vascular plant species. Nearly half of these species are endemic, and approximately 2400 of them are rare, threatened, or endangered (Myers et al. 2000). We selected the annual serpentine‐tolerant herb *Antirrhinum vexillocalyculatum* (Kellogg, also referred to as *Sairocarpus vexillocalyculatus* and called Wiry Snapdragon, Plantaginaceae, Figure S1) for this study because it is found on serpentine and non‐serpentine soil along the moisture cline in central California. Mediterranean climates such as the one in our study area are characterized by mild, wet winters and hot, dry summers. Therefore, effects of climate change may be particularly strong for *A. vexillocalyculatum* because it flowers late in the growing season. *Antirrhinum vexillocalyculatum* is an outcrossing species that is typically found growing on gravelly lower slopes of hillsides or rockslides, and individuals have been reported to occur approximately 55%–64% of the time on serpentine soils (Safford et al. 2005). It is native to California and has a wide range that spans east–west from the foothills of the Sierra Nevada mountains to the coast, and north–south from the Inner South Coast Ranges to the High North Coast Ranges (Calflora 2022; Jepson Flora Project 2022). It produces purple flowers that bloom sequentially from the bottom of a flowering stalk between June and August (Jepson eFlora 2022; Figure S1).

**FIGURE 1:**
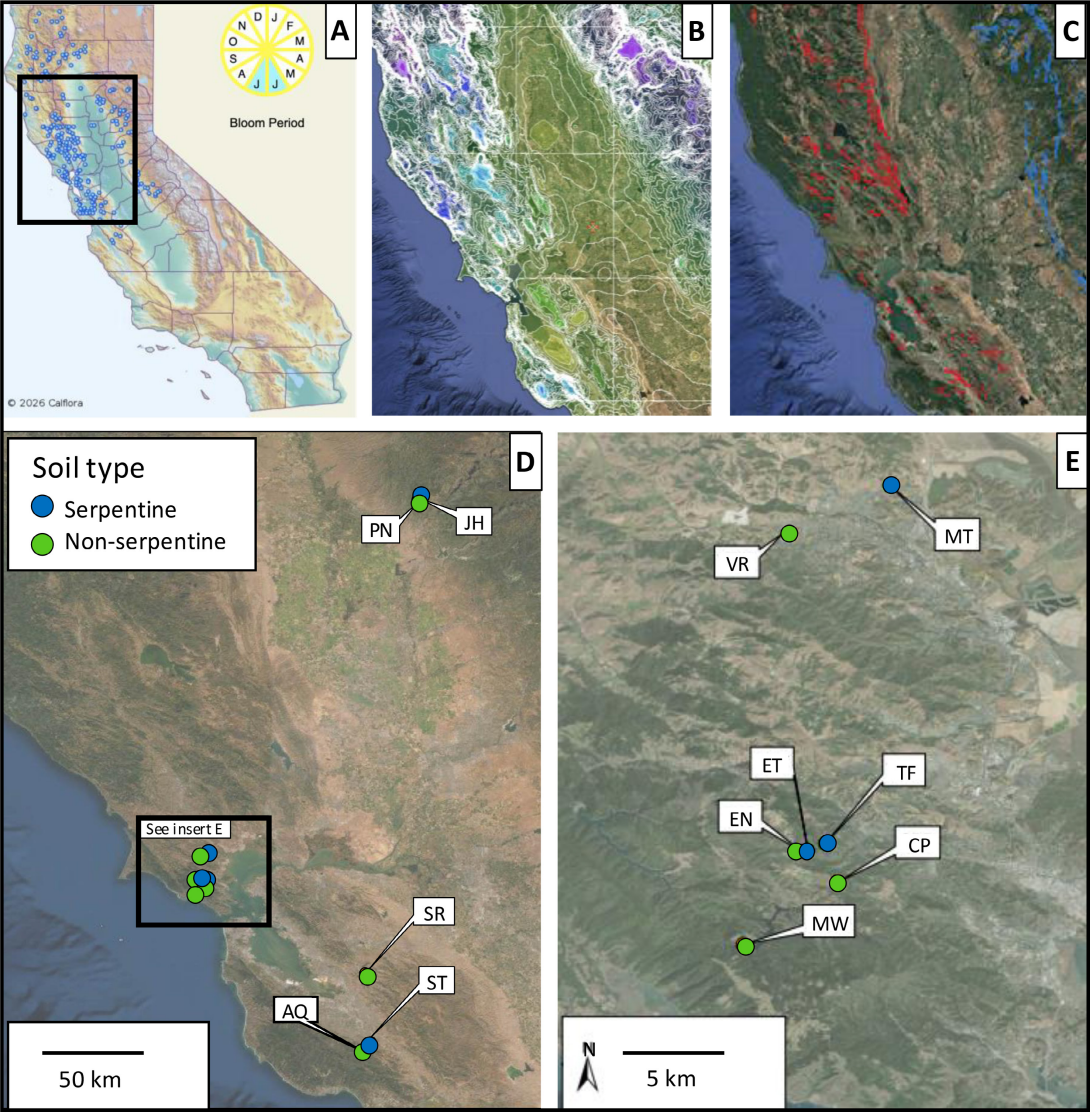
(A) Range of *Antirrhinum vexillocalyculatum* in California, USA. Blue points represent observation records. The rectangle reflects the sampling range of our study; (B) Mean annual precipitation (1971–2000) throughout our sampling range; (C) Serpentine areas within our sampling range. Blue and red outlines represent ultramafic rocks (mostly serpentinine) uplifted during the Late Proterozoic to Early Jurassic or Middle to Late Jurassic, respectively; (D) Map of study sites at which *Antirrhinum vexillocalyculatum* was sampled in northern California. Blue points represent serpentine populations and green points represent non‐serpentine populations; (E) Close‐up of Marin County sites within the black rectangle on (D). Data sources: Data for (A–C) was obtained from Calflora.org, accessed April 26, 2026, which uses data from the PRISM Climate Group for climatic data (prism.oregonstate.edu).

During the summer of 2020, we collected seeds from paired serpentine and non‐serpentine populations along the moisture cline in central California. We used iNaturalist.org and Calflora.org (accessed in April 2020) to identify serpentine and non‐serpentine populations, and we then selected populations that varied in their mean annual precipitation using data from the California Data Exchange Center's precipitation gauge. The non‐serpentine areas are made up primarily of Mollisol soils, which tend to be basic and have high organic matter content. In California, Mollisol soils tend to be Xeroll soil, which tends to be dry for extended periods in the summer but stores moisture above bedrock during the rest of the year (nrcs.usda.gov). We collected seeds at 11 sites that represented five population pairs and one additional non‐serpentine population (Table 1). Nine site pairs and the additional non‐serpentine site were located in the greater San Francisco Bay Area, and one pair was located in the western foothills of the Sierra Nevada, near Magalia, CA (Figure 1). Nine sites contained plants that were *A. vexillocalyculatum* ssp. *vexillocalyculatum* and the northernmost pair contained *A. vexillocalyculatum* ssp. *intermedium* , which is distinguished from ssp. *vexillocalyculatum* by having slightly longer nodes on the upper calyx, slightly longer corollas and the presence of some darker veins (Jepson eFlora, https://ucjeps.berkeley.edu/eflora/, accessed on February 12, 2025). At each site we removed 3–6 mature fruits from at least 30 individual plants, targeting plants that were at least 1 m apart from previously‐sampled plants. For each plant, we collected seeds into a separate labeled coin envelope, and we stored envelopes at room temperature until seeds were planted.

**TABLE 1:** Field sites in California; location description; paired site with the other soil type if present; soil type (Serp = serpentine soil, Non = non‐serpentine soil); whether the sites were used in the greenhouse study (GH), field study, or both; GPS coordinates; elevation; mean annual precipitation (MAP); mean spring precipitation (MSP); and mean annual temperature (MAT).

Site	Location	Pair	Soil	Study	Longitude	Latitude	Elev. (m)	MAP (mm)	MSP (mm)	MAT (°C)
ST	Santa Teresa Park, South San Jose, Santa Clara Co.	SR	Serp	Both	−121.80645	37.20402	136	598	145	15.3
SR	Sunol Regional Wilderness, Sunol, Alameda Co.	ST	Non	GH	−121.83172	37.53560	344	593	155	15
AQ	New Almaden Quicksilver Park, Santa Clara Co.	ST	Non	Field	−121.82706	37.17459	183	764	195	14.7
MT	Mt. Burdell, Novato, Marin Co.	VR	Serp	GH	−122.57939	38.12735	73	877	184	15.1
VR	Verissimo Hills, Novato, Marin Co.	MT	Non	GH	−122.62015	38.10795	124	992	209	15.1
EN	Elliot Nature Preserve, Fairfax, Marin Co.	ET	Non	GH	−122.61545	37.97881	66	1053	228	14.9
ET	Elliot Nature Preserve, Fairfax, Marin Co.	EN	Serp	GH	−122.61269	37.97903	108	1082	231	14.8
MW	Marin Municipal Water District, Kent Pump Lake, Marin Co.	—	Non	GH	−122.63902	37.94080	212	1153	242	14.3
TF	Toyon Fire Road, Fairfax, Marin Co.	CP	Serp	Both	−122.60565	37.98200	225	1172	242	14.7
CP	Concrete Pump Road, Fairfax, Marin Co.	TF	Non	Both	−122.60071	37.96617	157	1156	247	14.7
JH	Jordan Hill Road, Magalia, Butte Co.	PN	Serp	GH	−121.57166	39.82659	615	1516	393	14.6
PN	Pine Needle Drive, Magalia, Butte Co.	JH	Non	GH	−121.57946	39.81345	693	1566	406	14.7

### 2.2 Greenhouse Planting

We sowed field‐collected seeds in germination trays that we filled with Sunshine Mix #4 potting soil (Sungro Horticulture, Agwam, MA). We randomly selected 30 maternal families from each population and sowed 1–25 seeds from that maternal family. We cold‐stratified seeds for 10 days at 4°C and then placed germination trays in a greenhouse. The greenhouse was set to a 16‐h period of daylight, with a temperature target of 10°C–24°C, and humidity of 15%. Seedling emergence began 2 weeks after placement in the greenhouse. Five weeks after placement in the greenhouse, we transplanted seedlings into 4‐in pots and randomized the locations of individual plants with respect to geographic location in the greenhouse. Our final sample included 1469 plants in the greenhouse that were divided into trays containing approximately 18 plants each. Seedlings had been randomized prior to being placed into trays, so trays contained plants from different maternal families (mean 5.4 ± 0.26 plants per maternal family in the experiment). We watered plants liberally, via bottom watering, until the start of the differential watering experiment.

### 2.3 Climatic Data

We obtained local precipitation data from ClimateNA (https://climatena.ca/default), which downscales PRISM climatic data (Daly et al. 2008) and provides gridded monthly climate normal data (800 × 800 m) to scale‐free point locations. We used the time period of 1981–2010, and we used mean spring precipitation in statistical models (see below) because the amount of spring precipitation is particularly important for growth and reproduction of annual plants such as *A. vexillocalyculatum* in regions with Mediterranean climates such as central California (Robinson et al. 2013).

### 2.4 Greenhouse Common Garden

We randomly assigned 30 trays (492 plants) to be in a lower water (hereafter “drought”) treatment and assigned the rest to be in the higher water (hereafter “well‐watered” treatment (977 plants)). We began the differential watering study once approximately 40% of the plants had produced flower buds but none had flowered (see below for how we accounted for whether a plant had produced buds prior to this time in models). Each well‐watered tray received two liters of water twice weekly. We stopped watering the drought trays and waited until plants in those trays showed signs of wilting, which occurred after 11 days. They were watered at the next watering event and at every third watering thereafter. A subset of plants from this study were used in a different study that evaluated how differential watering affects floral reward to pollinators (Suni et al. 2023). We confirmed that being in the drought treatment decreased plant water content by assessing the relative water content of leaves (see Suni et al. 2023 for details and for an experimental timeline).

**FIGURE 2:**
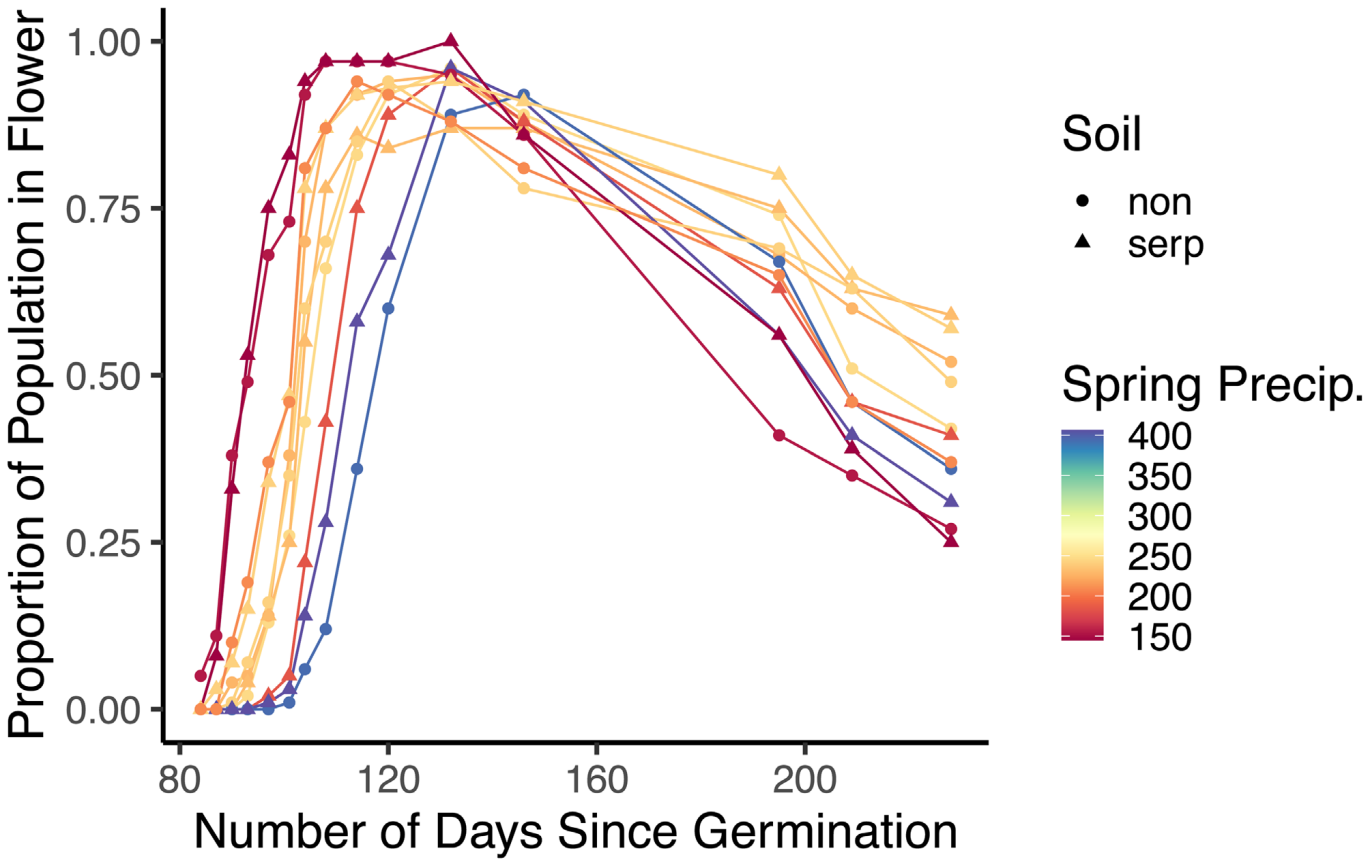
For each population, the proportion of plants in the well‐watered treatment that were flowering in the greenhouse over time. The *y*‐axis reflects the proportion of plants with open flowers at a given time point, with the number of days after germination reflected on the *x*‐axis. The decline in the proportion of plants with open flowers reflects the end of the reproductive phase and the transition to fruit production and senescence. Circles represent plants grown from seeds collected at non‐serpentine sites and triangles represent plants grown from seeds at serpentine sites. The color of each line reflects the spring precipitation of the site from which seeds were collected.

We recorded phenological data for 16 weeks post germination. We surveyed plants every 1–2 days and recorded the date that each plant produced a bud and first had an open flower. Once buds were observed, we recorded the number on each plant twice a week for 4 weeks. For two more weeks we recorded which plants budded for the first time. Once flowers opened, we recorded the number of open flowers per plant twice a week for 5 weeks, then once a week for another 3 weeks, and then once every other week for 8 weeks. We considered flowers to be open if the petal lips were reflexed and the corolla tube was open and had not yet shriveled or closed. For each individual plant, we quantified the highest floral abundance and the duration of flowering. We did this for a subset of plants that had completed flowering (and had no more buds) after 16 weeks, which represented a total of 1022 plants that included all populations (see Figure 2 for the proportion of plants from each population that were in flower over time). We recorded the date that each plant senesced.

### 2.5 Assessing Drivers of Reproductive Traits and Trait Divergence in the Greenhouse

The scale of inferences table below details the scale at which we seek to make inferences; the scales at which the treatment or factors of interest are applied; and the number of replicates for each level of treatment or factor.

**Table:** 

Scale of inference	Factor of interest	Scale at which factor of interest is applied	Number of replicates at the appropriate scale
Site	Precipitation	Site	11 sites that differ in precipitation
Site	Soil type	Site	Five serpentine and six non‐serpentine sites
Site pair	Phenological divergence	Site pair	Five site pairs that included one serpentine and one non‐serpentine site each
Individual	Treatment	Tray in greenhouse	30 drought trays and 60 well‐watered trays

We used linear mixed models implemented with the lme4 package to assess drivers of phenology (Bates et al. 2014) in R (R Core Team 2026). We ran separate models for response variables of interest that included: days to flowering onset, maximum flowers produced by individuals, days to maximum flowers produced by individual plants, flowering duration, days to peak flowering at the population level, and floral abundance at peak flowering. We included the mean spring precipitation of each site (MSP), soil type (serpentine or non‐serpentine), treatment (drought or well‐watered) as fixed factors; we included the maximum spring temperature (*Tmax_sp*) as a covariate to account for the effect of higher temperature on water use, although we omitted it when modeling the maximum number of flowers produced by individual plants because that model did not converge. We included the tray that each plant was in, the maternal family, the subspecies, and whether or not a plant had produced a bud prior to the initiation of the drought experiment as random effects. We included the interaction between soil type and precipitation in models because we were interested in evaluating whether the way that traits varied between soil types changed with precipitation. We included the interaction between soil type and treatment because we sought to understand whether the way that plants respond to drought differs according to native soil regime. We evaluated model fit by examining residuals to determine they met the assumption of normality using the plot function in R. To evaluate the significance of fixed factors we used the package lmerTest (Kuznetsova et al. 2017) to generate analysis of variance tables using Satterthwaite‐approximated degrees of freedom, and to calculate marginal (marg.) and conditional (cond.) *R* ^2^ values for models. We also calculated 95% confidence intervals (CI) for coefficients (denoted “Est.” in the results section) using the sjPlot package (Lüdecke et al. 2021).

To measure divergence across soil types and evaluate drivers of divergence, we calculated three metrics that included the absolute value of: the difference in the number of days to flowering onset, the date at which plants produced their maximum number of flowers (averaged across plants for each population in a pair prior to calculating the difference), and the difference in peak flowering date. We estimated divergence in peak flowering by identifying the day that the most flowers were observed at the population level, and by then taking the difference between those days for each population pair. We evaluated whether metrics of divergence vary with precipitation or are affected by differential watering using linear mixed models with subspecies as a random effect.

### 2.6 Assessing Drivers of Reproductive Traits and Trait Divergence in the Field

During the summer of 2021, we surveyed plants at four sites, including two serpentine and non‐serpentine pairs that receive different amounts of rainfall. The sites that received more rainfall were located in Marin County, CA, and they included sites TF and CP that were used in the greenhouse study (Table 1; Figure 1). The sites that received less rainfall were located in Santa Clara County, CA, and included the site ST that was used in the greenhouse study, as well as a nearby non‐serpentine site that was not used in the greenhouse study (site AQ), and that was chosen due to its proximity to ST (Table 1; Figure 1). We visited sites weekly for 9 weeks. During each site visit, we laid out a 10 m transect tape across the population and attempted to make it intersect the most *A. vexillocalyculatum* plants possible. We then walked along the transect and recorded every *A. vexillocalyculatum* plant that was within 10 cm of the transect tape. For each plant, we recorded the number of buds, number of flowers, number of fruits, and height. Some plants had already begun flowering when we began the field observations.

To evaluate drivers of reproductive phenology in the field, we used a linear model with floral abundance per plant as the dependent variable, precipitation regime (higher or lower), soil type, and date as fixed factors, and we assessed the significance of factors and their interactions using the summary function in R. We also characterized the date of peak flowering at the population level. We assessed divergence in reproductive traits and phenology using our transect data. First, we quantified the proportion of plants that were in flower at each site in a pair and calculated the difference in the proportion of plants that were in flower. Second, we quantified the difference in the mean number of flowers per plant between each site in a pair for each time we surveyed. We used *t*‐tests to test the hypothesis that divergence was greater at the wetter sites. We also visualized divergence using density plots of plants with more than one open flower using the *density* function in the ggplot2 (Wickham 2016) package in R.

Using phenological data from the field study, we evaluated the potential for gene flow between populations in each pair using a metric based on overlap in flowering phenology. This metric estimates the expected proportion of pollen that one population receives from its pair, assuming mating is random among individuals within and across populations (see equation 2 in Rivest et al. 2021). It ranges from 0 to 1 and provides an upper bound estimate of gene flow because it is based solely on flowering phenology and not on other potential reproductive isolating barriers. For two populations, *a* and *b*, the expected proportion of exchanged pollen into *b* from *a* is:

$${\mathrm{PGF}}_{a\to b}=\sum_{i=1}^{n}\frac{a_{i}}{a_{i}+b_{i}}*\frac{b_{i}}{\sum_{i=1}^{n}b_{i}}$$

Here, the proportion of total flowers from the donor population “*a*” are calculated at each time *i*: $\frac{a_{i}}{a_{i}+b_{i}}$. This is multiplied by a weighted sum at each time *i* with weights that are equal to the proportion of the number of open flowers in the receiving population “*b*” each day:

$$\frac{b_{i}}{\sum_{i=1}^{n}b_{i}}$$

Asymmetrical gene flow is likely common in nature (Tiffin et al. 2001; De Lafontaine and Bousquet 2017), so we applied this metric in both directions to assess the potential for asymmetries in gene flow. That is, we estimated the proportion of pollen that a non‐serpentine population “*b*” could receive from its serpentine pair “*a*” (${\mathrm{PGF}}_{a\to b}$) and we estimated the proportion of pollen that a serpentine population, “*a*”, could receive from its non‐serpentine pair, “*b*” (${\mathrm{PGF}}_{b\to a}$).

## 3 Results

### 3.1 Drivers of Reproductive Phenology in the Greenhouse Common Garden

**FIGURE 3:**
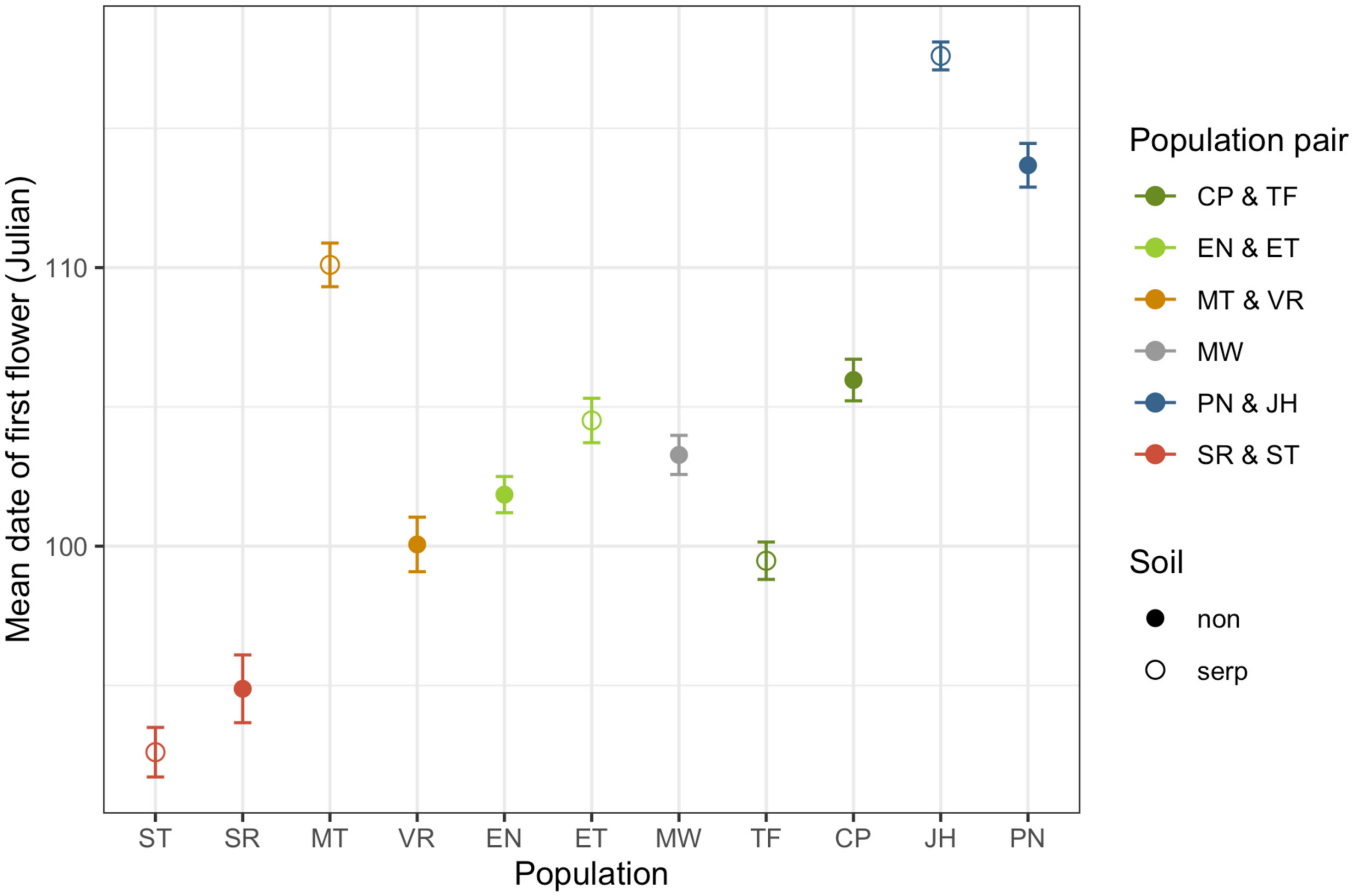
The mean date that plants in the greenhouse common garden flowered across individuals for each population in a pair. Colors represent different pairs (left to right in order of increasing precipitation), and shapes represent whether the population had serpentine (open circles) or non‐serpentine (filled circles) soil. Error bars represent standard errors.

Phenology was delayed in plants from wetter areas for all traits measured, and drought affected the expression of most traits. Flowering onset was later for plants from wetter sites (Est. = 0.03, df = 1.65, *p* = 0.049; CI = 0.02–0.05; Marg. *R* ^2^ = 0.084, Cond. *R* ^2^ = 0.7), earlier for plants from serpentine sites (Est. = −4.25, CI = −7.71 to −0.62, df = 229.46, *p* = 0.015), and serpentine plants showed a stronger delay along the precipitation cline than did non‐serpentine plants (Soil × precipitation Est. = −0.33, CI = 0.0035–0.028, df = 212.84, *p* = 0.01; Figures 2, 3, Figure S1). Drought delayed flowering onset (Est. = −1.22, CI = −2.59 to 0.15, df = 85.59, *p* = 0.017).

The date at which plants produced their maximum number of flowers was later for plants from wetter sites (Est. = 0.05, CI = 0.04–0.07, df = 300.76, *p* < 0.001), trended earlier for serpentine plants, although this difference was not significant (Est. = −3.41, CI −8.03–1.21, df = 270.61, *p* = 0.073), and was delayed by drought (Est. = −1.44, CI = −3.5–0.62, df = 88.38, *p* = 0.014; Marg. *R* ^2^ = 0.21).

The maximum number of flowers produced by individual plants was lower in those from areas of higher precipitation (Est. = −0.11, CI = −0.15 to −0.08, df = 275.94, *p* < 0.001; Marg. *R* ^2^ = 0.14, Cond. *R* ^2^ = 0.82; Figure S3). Drought did not affect the maximum number of flowers produced by individual plants (Est. = −1.839, CI = −4.01 to 0.23, df = 85.99, *p* = 0.2), nor did soil (Est. = −2.76, CI = −8.05 to 2.53, df = 256.21, *p* = 0.46).

Flowering duration was 6.9% shorter under the drought treatment (Mean well‐watered = 88 days, mean drought = 81.9 days, Est. = −3.84, CI = −7.9 to −0.95, df = 935.14, *p* < 0.001; Marg. *R* ^2^ = 0.057, Cond. *R* ^2^ = 0.13; Figure S4). There was a nonsignificant trend towards serpentine plants having higher plasticity in the extent to which flowering duration was reduced by drought (Est. = −7.02, CI = −14.07 to −0.02, *p* = 0.051, Figure S5; Soil est. = −6.39, CI = −21.42 to 8.63, df = 184.9). There was a nonsignificant trend towards flowering duration being shorter for plants from wetter areas (Est. = −0.07, CI = −0.14 to −0.01, df = 5.39, *p* = 0.068).

Peak flowering was earliest for control plants from the driest non‐serpentine site (April 16) and occurred on the same date for plants from most sites (April 21) except three sites high on the precipitation cline (Site MT well‐watered: May 7; Site PN drought: May 17; Site PN well‐watered & Site JH both treatments: May 24). Peak flowering date did not vary with soil, precipitation or treatment (Soil Est. = 1.95, −9.16 to 13.12, df = 14, *p* = 0.93; Precipitation Est. = 0.02, CI = −0.03 to 0.07, df = 14.7, *p* = 0.47; Treatment Est. = −0.17, CI = −4.07 to 3.74, df = 14, *p* = 0.36; Marg. *R* ^2^ = 0.013, Cond. *R* ^2^ = 0.96).

Floral abundance at peak flowering was 47% lower for plants in the drought treatment (Mean well‐watered = 848.6, Mean drought = 446.7, Est. = −425.17, CI = −692.49 to −157.84, df = 14, *p* < 0.001; Marg. *R* ^2^ = 0.19, Cond. *R* ^2^ = 0.8; Figure S6) and did not vary with soil or precipitation (Soil Est. = −320.85, CI = −1101.69 to 423.81, df = 14.01, *p* = 0.47; Precipitation Est. = 0.27, CI = −3.93 to 2.03, df = 10.5, *p* = 0.5).

### 3.2 Drivers of Divergence in Reproductive Phenology in the Greenhouse Common Garden

**FIGURE 4:**
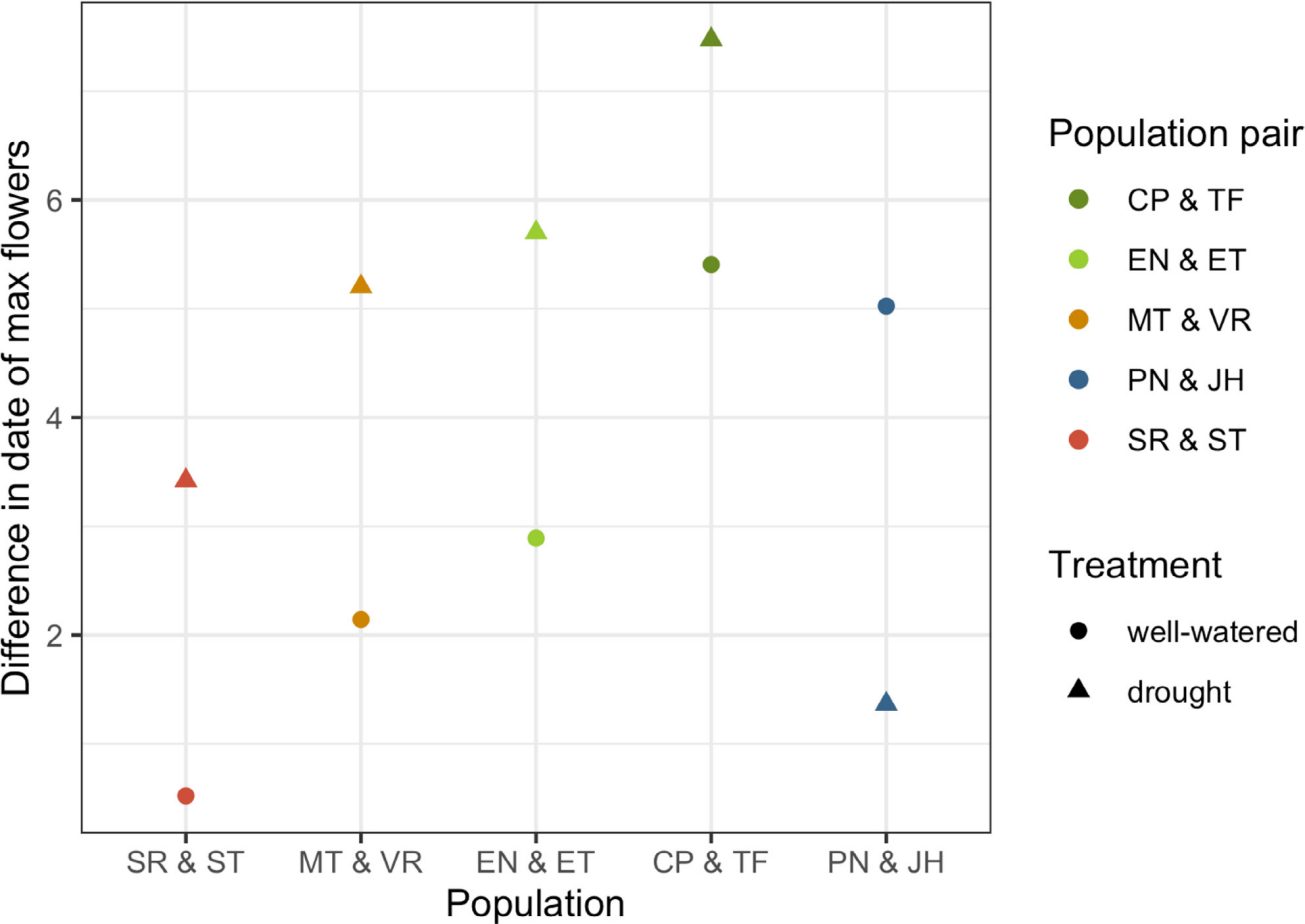
For each population pair ordered from lowest to highest precipitation (left to right), the number of days between the dates at which plants produced their maximum number of flowers in the greenhouse common garden. The four left most population pairs were *A. vexillocalyculatum* ssp. *vexillocalyculatum* and the right most pair was subspecies *intermedium*.

The difference in flowering onset did not vary with precipitation or water limitation (Precipitation Est. = −0.003, CI = −0.03–0.03, df = 6, *p* = 0.64; Treatment Est. = 2.37, CI = −8.51–13.26, df = 6, *p* = 0.73, Marg. *R* ^2^ = 0.051). The way that native precipitation regime influenced the difference between soil types in the date at which plants produced their maximum number of flowers varied across populations, such that it was later for plants from wetter sites and was increased by water limitation for all population pairs except the wettest pair, which represented *A. vexillocalyculatum* ssp. *intermedium* (Treatment est. = 8.28, CI = 4.89–11.66, df = 5, *p* = 0.0073; Precipitation Est. = 0.05, CI = 0.027–0.066, df = 5.45, *p* = 0.0083; Precipitation × Treatment Est. = −0.29, CI = −0.042 to −0.015, df = 5, *p* = 0.012; Marg. *R* ^2^ = 0.28, Cond *R* ^2^ = 0.98; Figure 4).

### 3.3 Field Study

Floral abundance was higher at drier sites (Est. = 27.64, CI = 21.4–33.87, df = 1635.5, *p* < 0.001), decreased over time (Est. = −0.02, CI = −0.039 to −0.00004, df = 323.5, *p* < 0.001), and declined more steeply at drier sites over time (Est. = −0.14, CI = −0.173 to −0.102, df = 848.1, *p* < 0.001, *R* ^2^ = 0.22). There was no difference in floral abundance between soil types (Est. = −4.16, CI = −11.0–2.7, df = 17.7, *p* = 0.27). Peak flowering at the population level was earliest at the wetter serpentine site (TF: June 1, AQ: June 3, ST: June 11, CP: June 15), while the date at which plants produced their maximum number of flowers (averaged across plants within populations) was earliest for the two serpentine sites and occurred on the same day (ST & TF: June 1, AQ: June 11, CP: June; Table S1; Figure S7).

**FIGURE 5:**
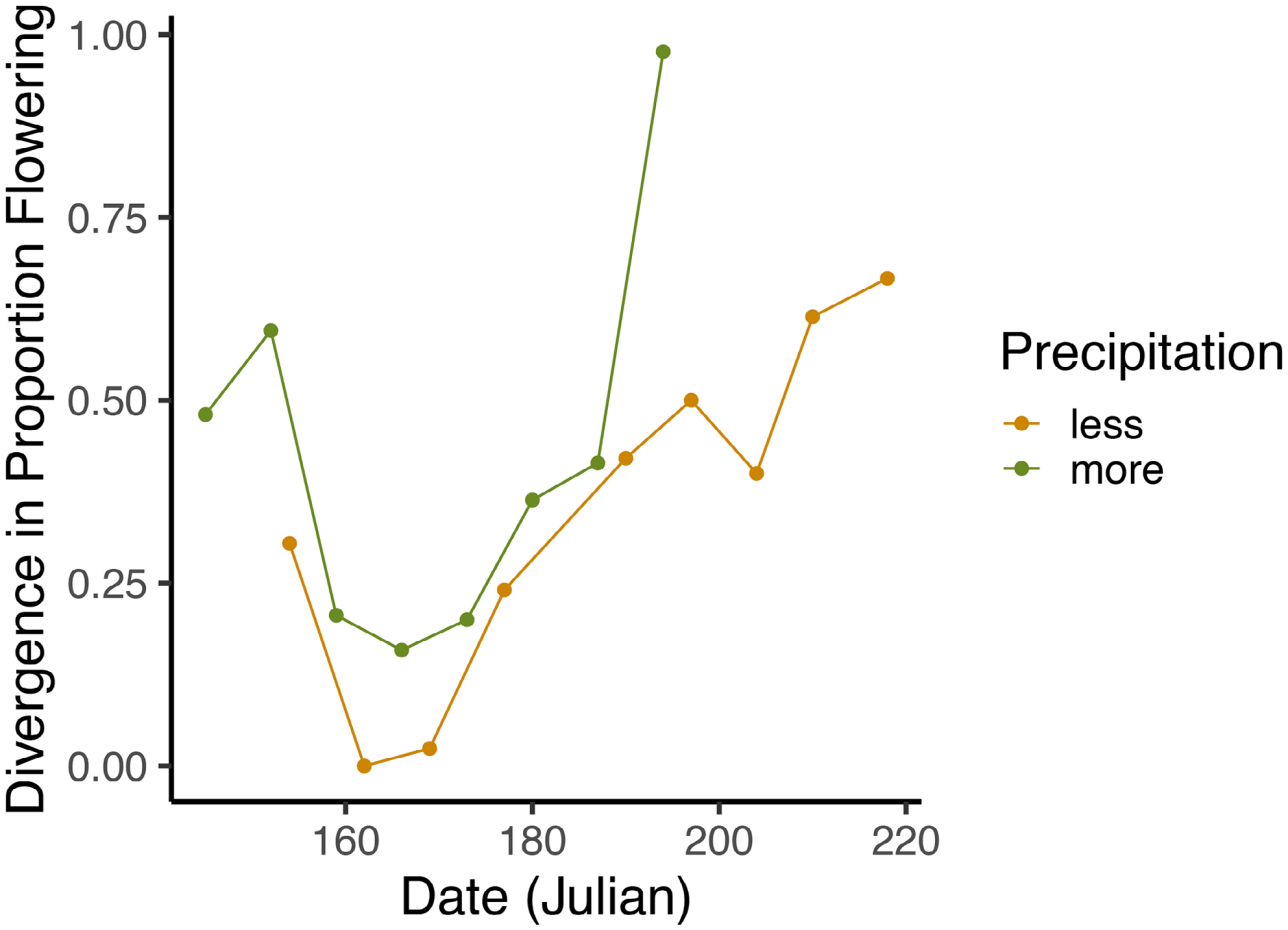
The divergence between serpentine and non‐serpentine field populations in the proportion of plants that were in flower assessed at site pairs that varied in precipitation. Values of points reflect the difference in the proportion across soil types (serpentine minus non‐serpentine). Green reflects the northern population pair (CP & TF) that receives more precipitation, and orange reflects the southern population pair (AQ & ST) that receives less precipitation. The displacement of points between lines that reflect more and less precipitation, as well as the difference in the number of sampling events between regions reflect the logistical effort required to monitor the four populations over time. The distance between the population pairs was 136 km, so each population within a pair was surveyed on the same day but pairs were surveyed on different days.

The difference between serpentine and non‐serpentine populations in the proportion of plants that were in flower was consistently greater in the wetter area (*t* = −2.24, CI = −Inf to −1.8, df = 5, *p* = 0.037, Figure 5). The difference in the mean number of flowers per plant was not greater in the wetter area (*t* = 3.24, CI = −Inf–2.5, df = 5, *p*‐value = 0.99). The divergence across soil types in density of plants that had more than one open flower was greater in the wetter area (Figure S8).

**FIGURE 6:**
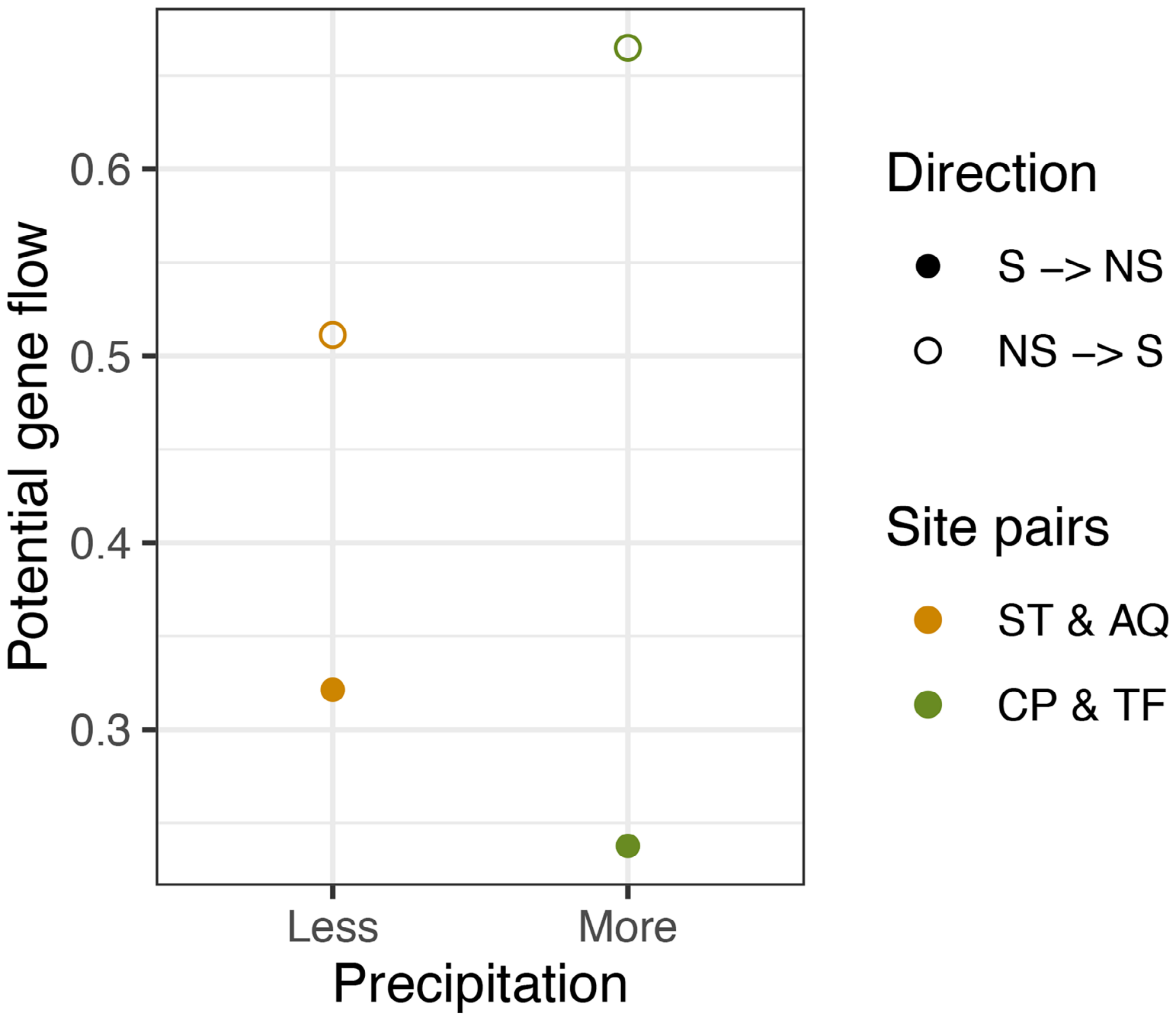
For two non‐serpentine—serpentine population pairs in regions with more and less precipitation, the relationship between the potential for gene flow from serpentine to non‐serpentine (filled circles), and non‐serpentine to serpentine (open circles), based on flowering abundance over time and assessed by field observations. Sites in the low precipitation region are in orange (SR & AQ) and sites in the higher precipitation region are in green (PN & JH).

The potential for gene flow ranged from 0.24 to 0.67 (Drier sites mean = 0.42; Wetter sites mean = 0.45). It was highest from non‐serpentine to serpentine sites in both wetter and drier areas, with the asymmetry being higher at wetter sites (Figure 6).

## 4 Discussion

Selection along environmental gradients can drive trait divergence in plant populations. We predicted that plants from drier sites and serpentine sites would have advanced reproductive phenology relative to those from non‐serpentine and wetter sites. Consistent with this prediction, flowering phenology was advanced for plants from drier areas in the greenhouse common garden, which is suggestive of local adaptation along the moisture cline (Dai et al. 2022). The genetic mechanisms underlying flowering onset in plants can include loci whose expression is dependent on the length of light that a plant experiences over 24 h (the photoperiod), ambient temperature (Osnato et al. 2022, and references therein), or soil moisture (Dai et al. 2022). In *Antirrhinum majus* , a related species that is commonly cultivated horticulturally, both photoperiod and temperature can affect flowering onset (Cremer et al. 1998), and our results suggest the additional mechanism of soil moisture as a strong driver of flowering in *A. vexillocalyculatum* . We also found some evidence of local adaptation to soil; soil provenance mediated the way precipitation affected flowering onset, and there were trends towards serpentine plants having earlier dates of maximum flower production and stronger reductions in flowering duration under drought. Results from our field observations suggest that soil is a strong driver of flowering phenology, and is perhaps more important than precipitation regime. The date at which plants produced their maximum number of flowers was earlier at serpentine sites across all sites examined (in both wetter and drier areas). The stronger effects of soil on phenology found for plants in the field versus greenhouse common garden suggest trait differences are mediated to some extent by plasticity, but the similarities between the common garden and field observations also suggest similar genetic architecture underlying most phenological traits examined in *A. vexillocalyculatum* across soil types.

It is interesting that our greenhouse common garden revealed a consistent increase in flowering onset from drier to wetter areas for non‐serpentine plants, while there was more variability in flowering onset across the precipitation gradient for serpentine plants. That is, the overall pattern of earlier flowering at serpentine sites was reversed for some pairs of sites, particularly in wetter areas. Past work has found that the direction of phenological shifts on serpentine soil is not consistent across species (Schneider 2017; Sianta and Kay 2021). For taxa that show accelerated flowering (Brady et al. 2005; Wright et al. 2006; Sianta and Kay 2019), this may be driven by serpentine soil being more xeric (Walker 1954; Kruckeberg 2002), which can trigger earlier flowering due to increased soil temperatures (Porto et al. 2024). Taxa that show delayed flowering on serpentine soil may be experiencing developmental limitations caused by a low‐nutrient environment (Zhang et al. 2023). The extent to which soil in serpentine areas is weathered, as well as the levels of heavy metals can vary across serpentine areas (Kazakou et al. 2010). Soil chemistry has been shown to affect phenology in other studies (Stanton et al. 2000; Brun et al. 2003; Ryser and Sauder 2006), so it would worthwhile to explore if the variability we found in the relationship between flowering onset across soil types along the precipitation gradient is driven by soil chemistry.

We found some support for the hypothesis that phenological divergence is higher between serpentine and non‐serpentine populations in wetter areas. In the greenhouse common garden, divergence in the date at which plants produced their maximum number of flowers was higher for plants from wetter areas, which was driven by plants from non‐serpentine soils in wetter areas producing their maximum flowers later than plants from serpentine sites. However, there was some variability in the relationship such that subspecies *intermedium* had phenotypic differences across soil types that were more similar to subspecies *vexillocalyculatum* in drier areas (Figure S9). In the field, divergence across soil types in the proportion of the population that was in flower was consistently higher at wetter sites. These results suggest that climatic productivity can drive phenological divergence across soil types, as has been found for growth‐related traits (Fernandez‐Going et al. 2013). Fernandez‐Going et al. (2013) showed that at the community level, non‐serpentine sites in wetter areas tended to contain plant species with traits that are associated with fast growth, such as high specific leaf area (SLA), leaf water content, and height, which may enable plants to compete with neighbors for light in communities with high density (Cornwell and Ackerly 2009). In contrast, Fernandez‐Going et al. (2013) found that serpentine sites in both wetter and drier areas tended to contain species with stress‐tolerant traits associated with a slower‐growing trait syndrome, which might enable plants to conserve resources in nutrient and water‐limited environments (Ordoñez et al. 2009). In our case, the earlier phenology we observed at both drier sites and serpentine sites is consistent with a life history strategy that prioritizes resource conservation over fast growth, and likely contributed to the observed phenological divergence we observed across soil types in wetter areas. That said, flowering distributions still overlapped to a large extent even for plants grown from seeds from areas with more precipitation, and also for the wetter pair of sites in the field. This suggests that the effect of climate on phenological divergence may be limited and may not result in gene flow being particularly restricted between serpentine and non‐serpentine populations, even in wetter areas. Indeed, the average potential for gene flow between serpentine and non‐serpentine sites in the field was similar for wetter and drier areas. In addition, our results warrant further investigation into differences among subspecies. It is possible that populations of subspecies *intermedium* in wetter areas would show a similar shift such that the date of maximum flower production is more delayed for non‐serpentine plants in the wetter part of the range, which we did not sample in this study.

While plants from both soil types showed high plasticity with regard to soil moisture, there was some evidence that serpentine plants showed greater plasticity under moisture limitation, as there was a trend towards drought reducing flowering duration more for serpentine plants. This could represent an adaptive response to drought, as plants on serpentine soil likely experience more xeric conditions relative to those on non‐serpentine, especially after the cessation of spring rains in our study area (Walker 1954; Kruckeberg 2002). Given costs of reproduction for plants (Dorken et al. 2025), we speculate that serpentine plants may have evolved to reallocate resources to seed development rather than maintain floral production under drought (Su et al. 2013). Unlike flowering duration, drought strongly reduced floral abundance at peak flowering similarly for serpentine and non‐serpentine plants, suggesting that plant reproduction will likely be compromised under future climates (Phillips et al. 2018; Kuppler et al. 2021).

We characterized the potential for gene flow across soil types under drought and non‐drought conditions in an attempt to understand the potential for adaptive gene flow across soil types under future climates. Our results suggest that gene flow from serpentine to non‐serpentine populations could occur and be adaptive, but that effects might not be particularly strong. Serpentine plants flowered earlier and showed somewhat greater plasticity in how flowering duration responded to water limitation, consistent with the notion that they may be better able to escape or avoid drought than non‐serpentine plants. However, gene flow from non‐serpentine to serpentine populations was higher than in the other direction. Furthermore, both serpentine and non‐serpentine plants had shortened flowering durations under moisture limitation, which suggests the presence of a shared, ancestral water‐conservation strategy that could have evolved because flowers lose water readily relative to other plant tissues (Nobel 1977). Therefore, given the similarities in drought response across soil types and the lower likelihood of gene flow from serpentine to non‐serpentine populations, we speculate that adaptive gene flow from serpentine to non‐serpentine populations may not be a particularly strong force in this system. It is worth noting that our measure of gene flow was based on phenological overlap, which provides an idealized upper limit and may overestimate true connectivity. Gene flow is also determined by pollinator foraging behavior, the spatial distance between populations, and potential post‐mating barriers (Baack et al. 2015). It would therefore be worthwhile for future studies to investigate reproductive isolation among serpentine and non‐serpentine populations, as well as the extent to which there is genetic connectivity across soil types for *A. vexillocalyculatum* .

Overall, our extensive phenological tracking of plants grown from seeds collected from serpentine and non‐serpentine populations along a moisture cline in a greenhouse common garden revealed evidence for local adaptation to moisture regime and weaker effects of soil provenance. However, soil did mediate the way flowering phenology varied along the climatic cline, particularly in terms of flowering onset. Phenological divergence was higher across soil types in wetter areas, but overlap in flowering distributions suggests ecotypes of *A. vexillocalyculatum* are only in incipient stages of divergence and that gene flow may be an important force in this system, particularly from non‐serpentine to serpentine populations.

## Author Contributions

**Nicole Ibañez:** conceptualization (equal), data curation (equal), formal analysis (equal), funding acquisition (equal), investigation (equal), methodology (equal), project administration (equal), visualization (equal), writing – original draft (supporting), writing – review and editing (supporting). **John Paul:** conceptualization (equal), project administration (equal), resources (equal), supervision (equal), validation (equal), writing – original draft (supporting), writing – review and editing (supporting). **Adelaide Ergastolo:** data curation (equal), investigation (equal), methodology (equal), writing – review and editing (supporting). **Sevan Suni:** conceptualization (equal), data curation (equal), formal analysis (lead), funding acquisition (equal), investigation (equal), methodology (equal), project administration (equal), resources (equal), software (lead), supervision (equal), validation (equal), visualization (equal), writing – original draft (lead), writing – review and editing (equal).

## Funding

This work was supported by a grant to S. Suni from the Faculty Development Fund at the University of San Francisco, San Francisco, CA, USA, and by an Award of Merit granted to N. Ibañez from the Marin Chapter of the California Native Plant Society.

## Conflicts of Interest

The authors declare no conflicts of interest.

## Supporting information


**Figure S1:** Photographs of *Antirrhinum vexillocalyculatum* taken in the field at serpentine (top left and bottom middle) and non‐serpentine (bottom right) sites, and in the greenhouse (top right and bottom left).
**Figure S2:** Date of first flower for populations of plants grown from seeds collected on serpentine (“serp,” blue) and non‐serpentine (“non,” green) soil along a precipitation gradient. Plots represent boxplots and points are actual data points that have been jittered for clarity.
**Figure S3:** Maximum number of flowers for populations of plants grown from seeds collected on serpentine (“serp,” blue) and non‐serpentine (“non,” green) soil along a precipitation gradient. Plots represent boxplots and points are actual data points that have been jittered for clarity.
**Figure S4:** The relationship between precipitation and mean flowering duration of plants grown from seeds collected from serpentine (triangles) and non‐serpentine (circles) sites and grown in a greenhouse common garden. Points represent populations that are separated by treatment. Blue represents well‐watered plants and orange represents water limited plants. Black arrows show the direction of the change between well‐watered and water‐limited groups.
**Figure S5:** Predicted results from models assessing the effects of soil type and water limitation on flowering duration. The *y*‐axis reflects the mean total number of days plants grown from seeds collected from different soil types were in flower. Orange reflects water‐limited plants and blue reflects well‐watered plants. There was a trend towards serpentine plants reducing their flowering duration more under drought than non‐serpentine plants.
**Figure S6:** Mean floral abundance on each sampling date for each population from the greenhouse common garden experiment. Blue points represent seeds collected from serpentine sites (top row) and green points represent seeds collected at non‐serpentine sites (bottom row). Circles and triangles represent plants in the well‐watered and water‐limited treatments, respectively. Sites are in order of increasing precipitation from left to right, and site pairs are in the same column. From left to right the serpentine – non‐serpentine pairs are: ST & SR, MT & VR, ET & EN, TF & CP, and JH & PN (See Table 1 for additional site information).
**Figure S7:** The number of flowers on individual plants that were surveyed at two pairs of field sites that had serpentine (triangles) or non‐serpentine soil (circles). Green points reflect the northern population pair (CP & TF) that receives more precipitation, and orange points reflect the southern population pair (AQ & ST) that receives less precipitation. Points represent individual plants and darker shades represent overlapping points. Plants in the drier region produced more flowers than plants in the wetter region. The distance between the population pairs was 136 km, so each population within a pair was generally surveyed on the same day and regions were surveyed on different days.
**Figure S8:** Kernal density plots of plants with more than one flower at two population pairs in the field that vary in precipitation, over time. The northern sites (CP & TF) receive more precipitation and the southern sites (AQ & ST) receive less precipitation. Green and blue reflect non‐serpentine and serpentine soil, respectively.
**Figure S9:** For each population pair, the average date at which plants in the well‐watered treatment produced their maximum number of flowers. Population pairs are colored in rainbow order based on their precipitation, with red representing lower and blue representing higher precipitation. The right most populations in blue represent subspecies *intermedium* while all populations to the left on the *x*‐axis are subspecies *vexillocalyculatum*.
**Table S1:** For each time that sites in two serpentine – non‐serpentine site pairs were visited, the soil type, region in the San Francisco Bay Area, precipitation regime, site name, date visited, total number of flowers across all plants, and mean number of flowers per plant.

## Data Availability

The data and code used in analyses are available in the Zenodo online data repository: https://zenodo.org/records/19952980.
