# Supplementary material for: Effects of Climate and Water Limitation on Reproductive Traits and Trait Divergence Across Soil Boundaries in a Serpentine‐Tolerant Annual Herb
Source: Ecol Evol. 2026 Jun 23;16(6):e73785. doi: 10.1002/ece3.73785 (PMC13290394; doi:10.1002/ece3.73785)
Supplement: Supplementary file 1 — Figure S1: Photographs of Antirrhinum vexillocalyculatum taken in the field at serpentine (top left and bottom middle) and non‐serpentine (bottom right) sites, and in the greenhouse (top right and bottom left). Figure S2: Date of first flower for populations of plants grown from seeds collected on serpentine (“serp,” blue) and non‐serpentine (“non,” green) soil along a precipitation gradient. Plots represent boxplots and points are actual data points that have been jittered for clarity. Figure S3: Maximum number of flowers for populations of plants grown from seeds collected on serpentine (“serp,” blue) and non‐serpentine (“non,” green) soil along a precipitation gradient. Plots represent boxplots and points are actual data points that have been jittered for clarity. Figure S4: The relationship between precipitation and mean flowering duration of plants grown from seeds collected from serpentine (triangles) and non‐serpentine (circles) sites and grown in a greenhouse common garden. Points represent populations that are separated by treatment. Blue represents well‐watered plants and orange represents water limited plants. Black arrows show the direction of the change between well‐watered and water‐limited groups. Figure S5: Predicted results from models assessing the effects of soil type and water limitation on flowering duration. The y‐axis reflects the mean total number of days plants grown from seeds collected from different soil types were in flower. Orange reflects water‐limited plants and blue reflects well‐watered plants. There was a trend towards serpentine plants reducing their flowering duration more under drought than non‐serpentine plants. Figure S6: Mean floral abundance on each sampling date for each population from the greenhouse common garden experiment. Blue points represent seeds collected from serpentine sites (top row) and green points represent seeds collected at non‐serpentine sites (bottom row). Circles and triangles represent plants in the wel [file ECE3-16-e73785-s001.pdf]

Supplementary information for: *Effects of climate and water limitation on reproductive traits and trait divergence across soil boundaries in a serpentine-tolerant annual herb*

Table of Contents

Table S1……Page 2

Figure S1………Page 3

Figure S2………Page 4

Figure S3………Page 5

Figure S4………Page 6

Figure S5………Page 7

Figure S6………Page 8

Figure S7………Page 9

Figure S8………Page 10

Figure S9………Page 11

Supplementary information for: *Effects of climate and water limitation on reproductive traits and trait divergence across soil boundaries in a serpentine-tolerant annual herb*

Soil	Region	Climate	Site	Date	Total flowers	Mean flowers
Non-serpentine	North	Wetter	CP	5/25/21	41	0.5
Non-serpentine	North	Wetter	CP	6/1/21	41	1.0
Non-serpentine	North	Wetter	CP	6/8/21	61	1.8
Non-serpentine	North	Wetter	CP	6/15/21	104	1.9
Non-serpentine	North	Wetter	CP	6/22/21	24	0.6
Non-serpentine	North	Wetter	CP	6/29/21	21	0.5
Non-serpentine	North	Wetter	CP	7/6/21	6	0.2
Non-serpentine	North	Wetter	CP	7/13/21	4	0.1
Non-serpentine	North	Wetter	CP	7/20/21	0	0.0
Serpentine	North	Wetter	TF	5/27/21	15	1.5
Serpentine	North	Wetter	TF	6/1/21	39	2.8
Serpentine	North	Wetter	TF	6/8/21	24	1.4
Serpentine	North	Wetter	TF	6/15/21	11	0.8
Serpentine	North	Wetter	TF	6/22/21	10	1.0
Serpentine	North	Wetter	TF	6/29/21	5	1.3
Serpentine	North	Wetter	TF	7/6/21	1	0.5
Serpentine	North	Wetter	TF	7/13/21	3	1.5
Non-serpentine	South	Drier	AQ	6/3/21	196	8.5
Non-serpentine	South	Drier	AC	6/11/21	136	9.1
Non-serpentine	South	Drier	AC	6/18/21	73	5.2
Non-serpentine	South	Drier	AC	6/26/21	27	1.4
Non-serpentine	South	Drier	AC	7/1/21	4	0.5
Non-serpentine	South	Drier	AC	7/9/21	18	1.3
Non-serpentine	South	Drier	AC	7/16/21	0	0.0
Non-serpentine	South	Drier	AC	7/23/21	0	0.0
Non-serpentine	South	Drier	AC	7/29/21	1	0.1
Non-serpentine	South	Drier	AC	8/6/21	0	0.0
Serpentine	South	Drier	ST	6/1/21	54	10.8
Serpentine	South	Drier	ST	6/11/21	86	7.2
Serpentine	South	Drier	ST	6/18/21	37	2.5
Serpentine	South	Drier	ST	6/26/21	24	3.4
Serpentine	South	Drier	ST	7/9/21	47	5.2
Serpentine	South	Drier	ST	7/16/21	18	1.8
Serpentine	South	Drier	ST	7/23/21	5	1.0
Serpentine	South	Drier	ST	7/29/21	11	1.6
Serpentine	South	Drier	ST	8/6/21	4	1.3

Table S1. For each time that sites in two serpentine – non-serpentine site pairs were visited, the soil type, region in the San Francisco Bay Area, precipitation regime, site name, date visited, total number of flowers across all plants, and mean number of flowers per plant.

Supplementary information for: *Effects of climate and water limitation on reproductive traits and trait divergence across soil boundaries in a serpentine-tolerant annual herb*

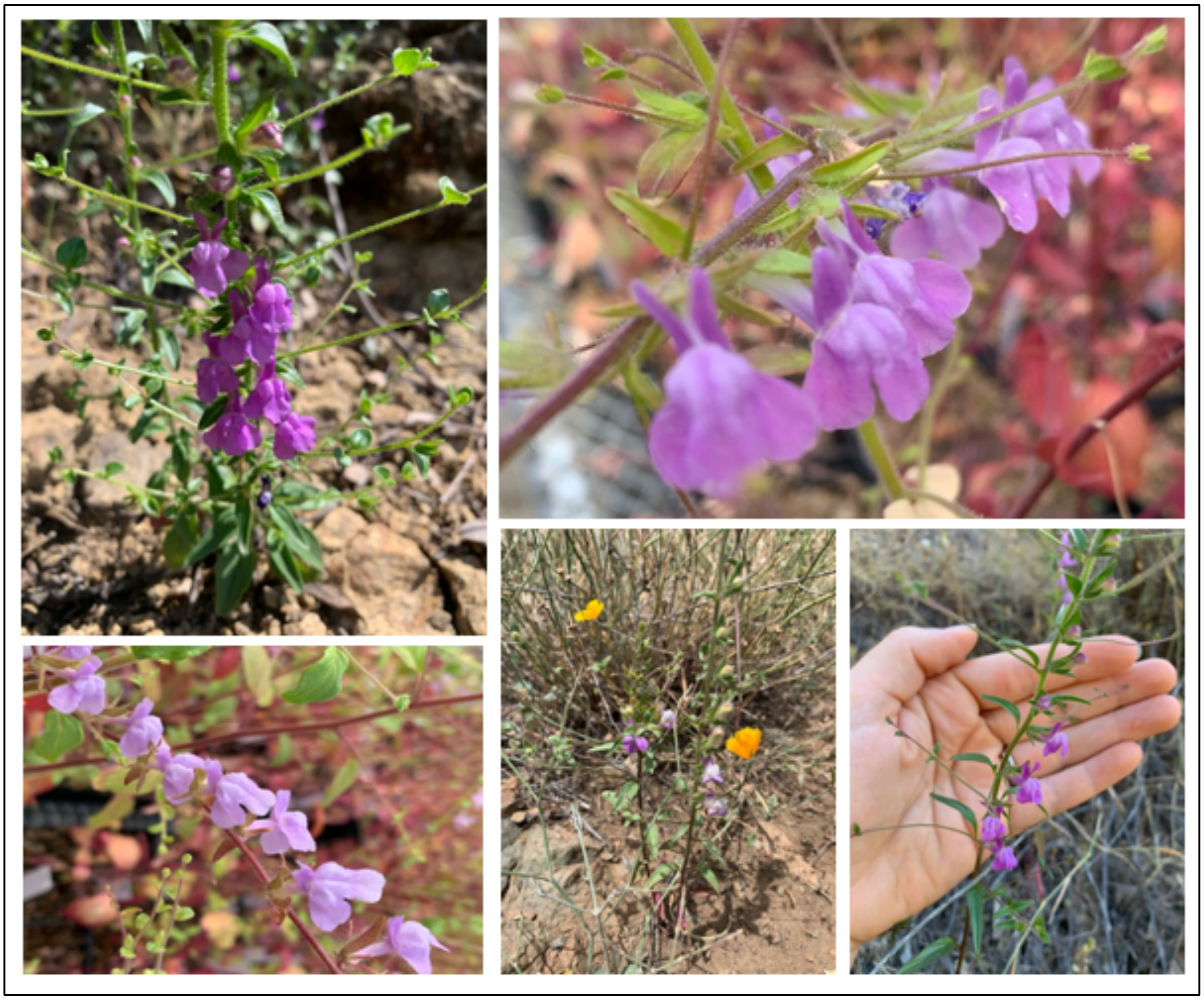

Figure S1. Photographs of *Antirrhinum vexillocalyculatum* taken in the field at serpentine (top left and bottom middle) and non-serpentine (bottom right) sites, and in the greenhouse (top right and bottom left).

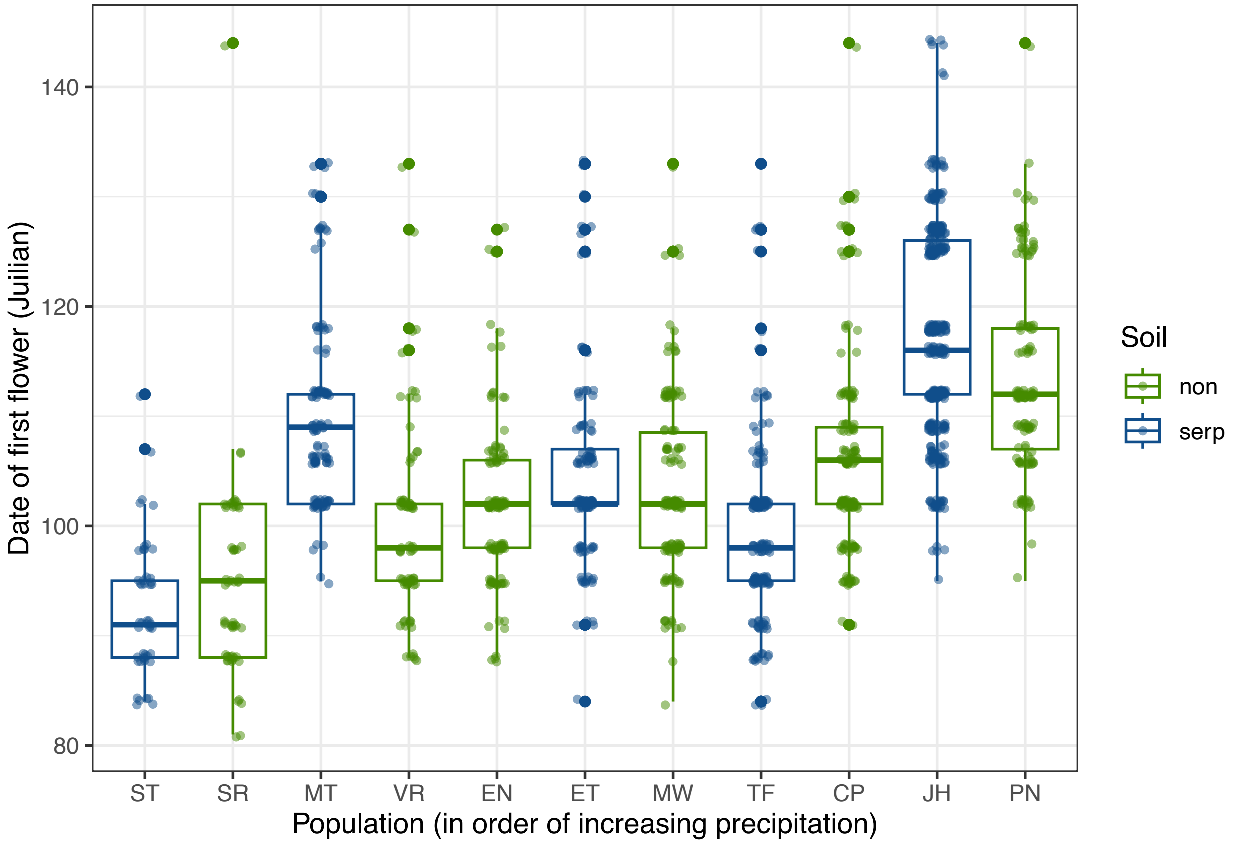

Fig. S2. Date of first flower for populations of plants grown from seeds collected on serpentine ("serp", blue) and non-serpentine ("non", green) soil along a precipitation gradient. Plots represent boxplots and points are actual data points that have been jittered for clarity.

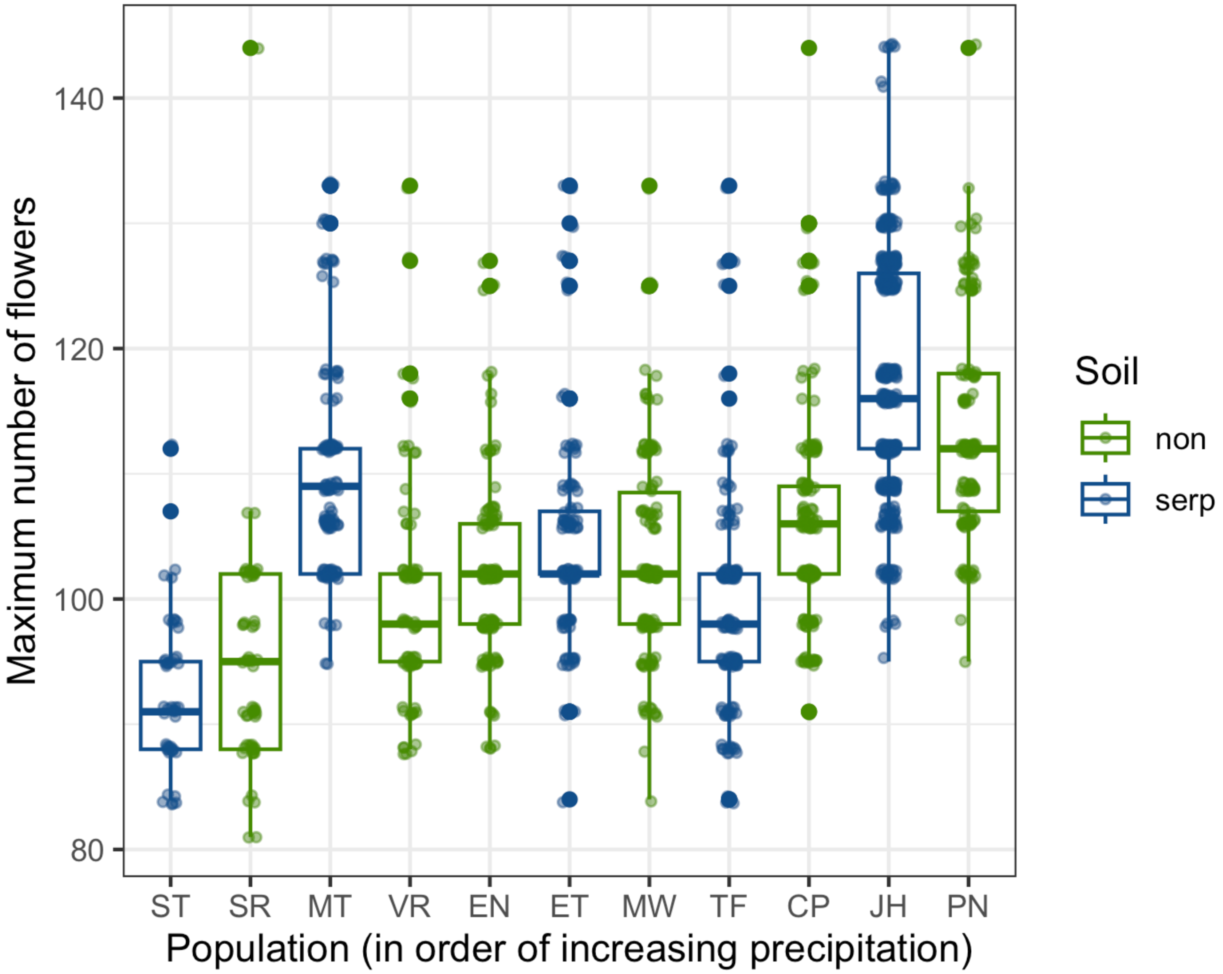

Fig. S3. Maximum number of flowers for populations of plants grown from seeds collected on serpentine (“serp”, blue) and non-serpentine (“non”, green) soil along a precipitation gradient. Plots represent boxplots and points are actual data points that have been jittered for clarity.

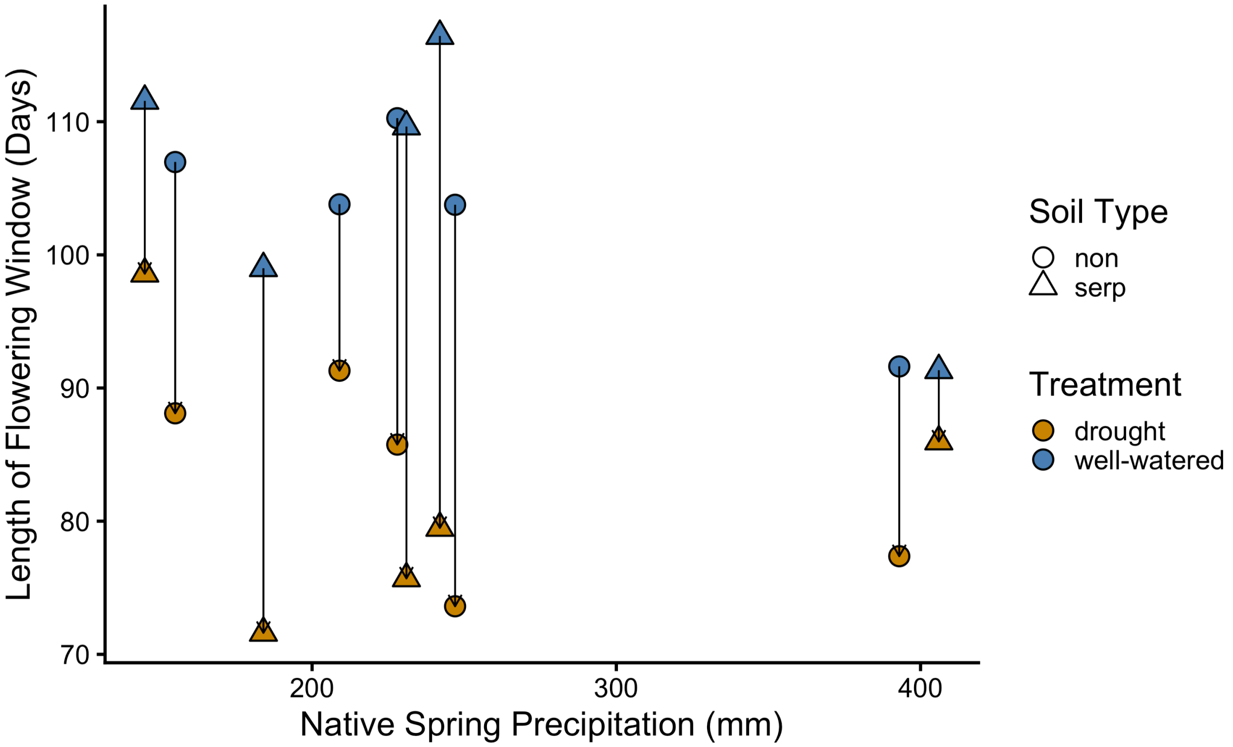

Fig. S4. The relationship between precipitation and mean flowering duration of plants grown from seeds collected from serpentine (triangles) and non-serpentine (circles) sites and grown in a greenhouse common garden. Points represent populations that are separated by treatment. Blue represents well-watered plants and orange represents water-limited plants. Black arrows show the direction of the change between well-watered and water-limited groups.

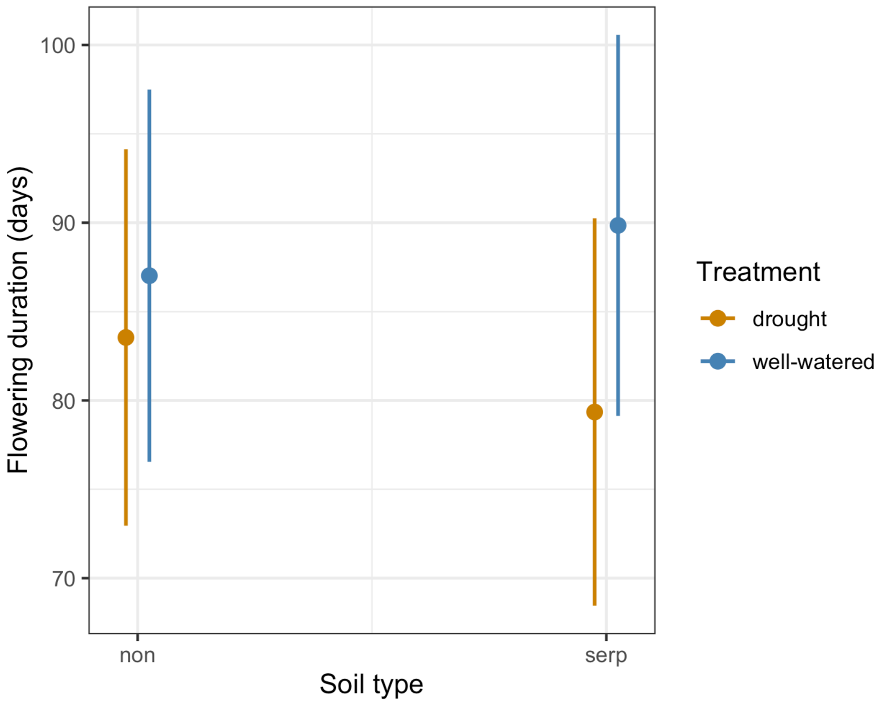

Fig. S5. Predicted results from models assessing the effects of soil type and water limitation on flowering duration. The y-axis reflects the mean total number of days plants grown from seeds collected from different soil types were in flower. Orange reflects water-limited plants and blue reflects well-watered plants. There was a trend towards serpentine plants reducing their flowering duration more under drought than non-serpentine plants.

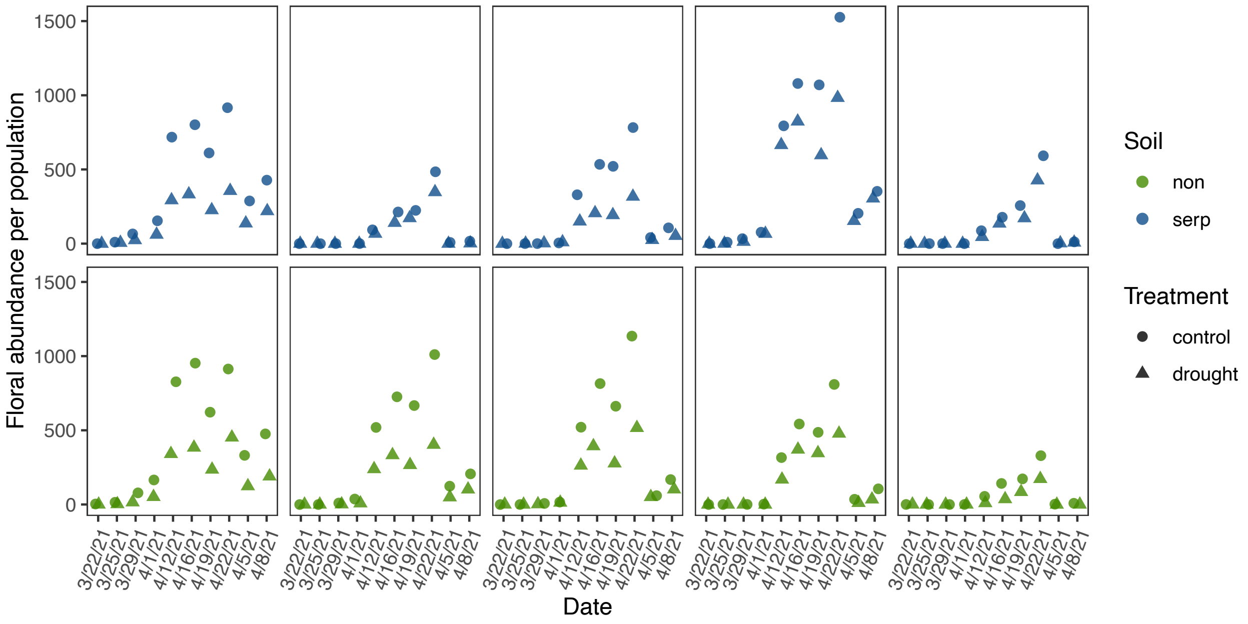

Fig. S6. Mean floral abundance on each sampling date for each population from the greenhouse common garden experiment. Blue points represent seeds collected from serpentine sites (top row) and green points represent seeds collected at non-serpentine sites (bottom row). Circles and triangles represent plants in the well-watered and water-limited treatments, respectively. Sites are in order of increasing precipitation from left to right, and site pairs are in the same column. From left to right the serpentine – non-serpentine pairs are: ST & SR, MT & VR, ET & EN, TF & CP, and JH & PN (See Table 1 for additional site information).

Supplementary information for: *Effects of climate and water limitation on reproductive traits and trait divergence across soil boundaries in a serpentine-tolerant annual herb*

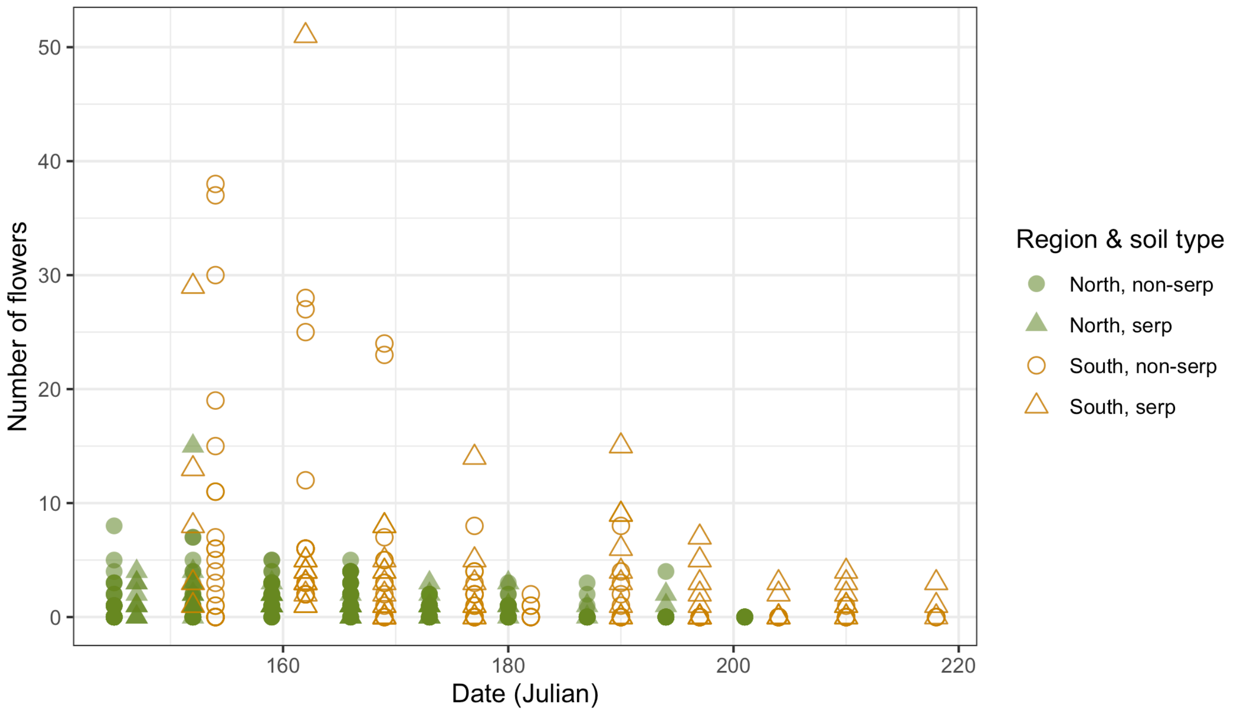

Fig. S7. The number of flowers on individual plants that were surveyed at two pairs of field sites that had serpentine (triangles) or non-serpentine soil (circles). Green points reflect the northern population pair (CP & TF) that receives more precipitation, and orange points reflect the southern population pair (AQ & ST) that receives less precipitation. Points represent individual plants and darker shades represent overlapping points. Plants in the drier region produced more flowers than plants in the wetter region. The distance between the population pairs was 136 km, so each population within a pair was generally surveyed on the same day and regions were surveyed on different days.

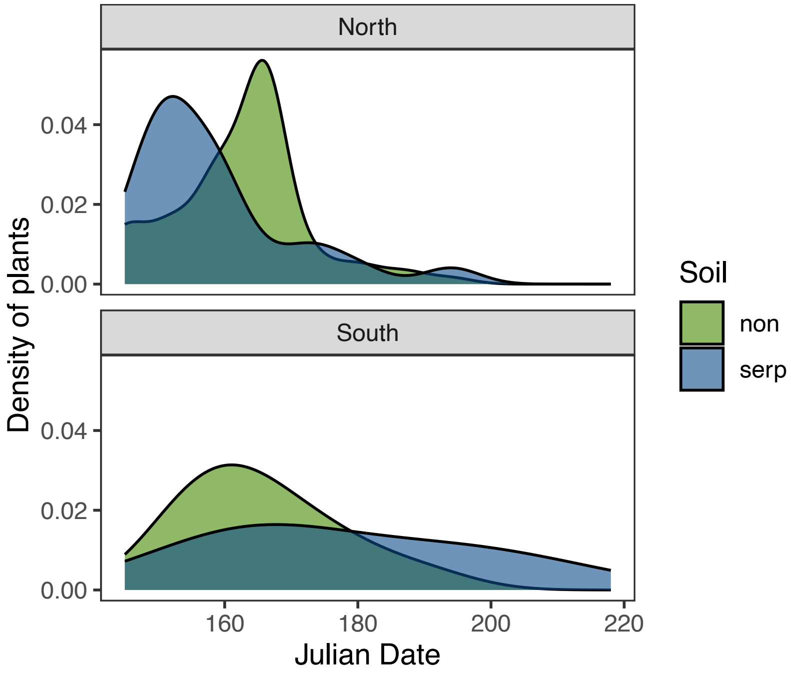

Fig. S8. Kernal density plots of plants with more than one flower at two population pairs in the field that vary in precipitation, over time. The northern sites (CP & TF) receive more precipitation and the southern sites (AQ & ST) receive less precipitation. Green and blue reflect non-serpentine and serpentine soil, respectively.

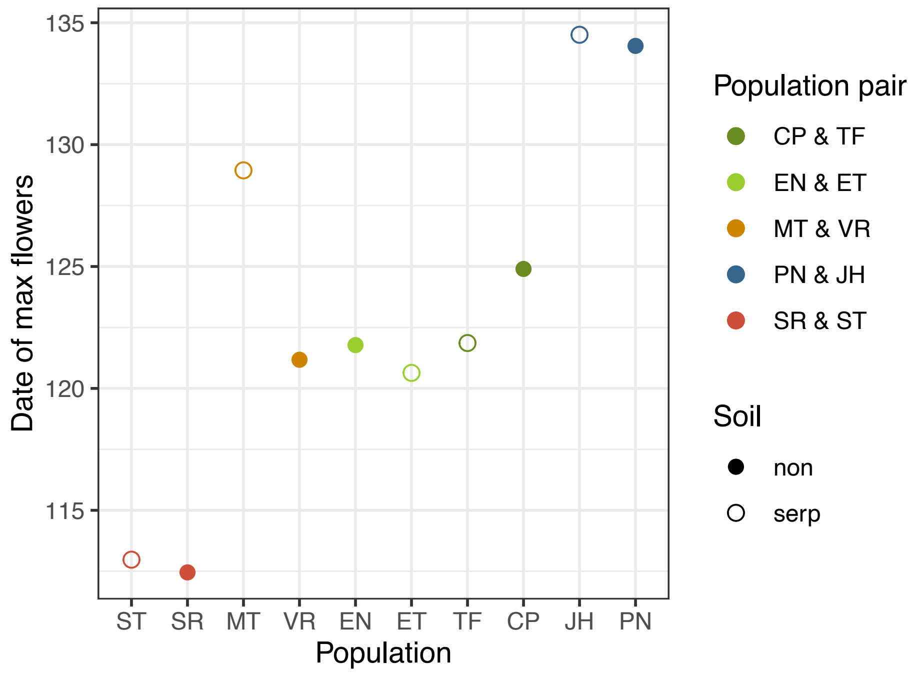

Fig. S9. For each population pair, the average date at which plants in the well-watered treatment produced their maximum number of flowers. Population pairs are colored in rainbow order based on their precipitation, with red representing lower and blue representing higher precipitation. The right most populations in blue represent subspecies *intermedium* while all populations to the left on the x-axis are subspecies *vexillocalyculatum*.
